# Fabrication of Concave Microwells and Their Applications in Micro-Tissue Engineering: A Review

**DOI:** 10.3390/mi13091555

**Published:** 2022-09-19

**Authors:** Weijin Guo, Zejingqiu Chen, Zitao Feng, Haonan Li, Muyang Zhang, Huiru Zhang, Xin Cui

**Affiliations:** 1 Department of Biomedical Engineering, Shantou University, Shantou 515063, China; 2 Department of Biology, Shantou University, Shantou 515063, China; 3 Department of Electrical Engineering, Shantou University, Shantou 515063, China; 4 Guangdong Foshan Lianchuang Graduate School of Engineering, Foshan 528311, China; 5 Key Laboratory of Biomaterials of Guangdong Higher Education Institutes, Department of Biomedical Engineering, Jinan University, Guangzhou 510632, China

**Keywords:** lithography, etching, photoresist reflow, CNC milling, 3D printing, surface tension, spheroid, organoid, embryoid, cellular behavior

## Abstract

At present, there is an increasing need to mimic the in vivo micro-environment in the culture of cells and tissues in micro-tissue engineering. Concave microwells are becoming increasingly popular since they can provide a micro-environment that is closer to the in vivo environment compared to traditional microwells, which can facilitate the culture of cells and tissues. Here, we will summarize the fabrication methods of concave microwells, as well as their applications in micro-tissue engineering. The fabrication methods of concave microwells include traditional methods, such as lithography and etching, thermal reflow of photoresist, laser ablation, precision-computerized numerical control (CNC) milling, and emerging technologies, such as surface tension methods, the deformation of soft membranes, 3D printing, the molding of microbeads, air bubbles, and frozen droplets. The fabrication of concave microwells is transferring from professional microfabrication labs to common biochemical labs to facilitate their applications and provide convenience for users. Concave microwells have mostly been used in organ-on-a-chip models, including the formation and culture of 3D cell aggregates (spheroids, organoids, and embryoids). Researchers have also used microwells to study the influence of substrate topology on cellular behaviors. We will briefly review their applications in different aspects of micro-tissue engineering and discuss the further applications of concave microwells. We believe that building multiorgan-on-a-chip by 3D cell aggregates of different cell lines will be a popular application of concave microwells, while integrating physiologically relevant molecular analyses with the 3D culture platform will be another popular application in the near future. Furthermore, 3D cell aggregates from these biosystems will find more applications in drug screening and xenogeneic implantation.

## 1. Introduction

As an emerging technology, organ-on-a-chip has attracted increasing attention from researchers in different fields [1]. Organ-on-a-chip technology can help researchers to better understand the physiology of different organs, and also greatly benefit the process of drug development by saving both time and cost in drug testing [2]. Huh et al. built a lung model on a chip to investigate the influence of respiration on alveolus cells and mimic their responses to nanoparticulates [3]. Agarwal et al. developed a microfluidic heart model on a chip to study the effect of drug on cardiac contractility in a high-throughput way [4]. Deng et al. established a microfluidic liver model to explore the hepatoprotective effect of three hepatoprotectants (tiopronin, bifendatatum, and glycyrrhizinate) [5]. Kim et al. used a microfluidic gut model to study the combined influence of peristalsis and microbiome on the growth and inflammation of bacteria in the human intestine [6]. A microfluidic human kidney model was used to investigate the nephrotoxicity of gentamicin [7]. A microfluidic model was developed to mimic the drug (glyburide–a gestational diabetes drug) transportation through the human placental barrier [8]. At present, researchers are trying to build a multiorgan-on-a-chip model or even a human-on-a-chip model to deepen the understanding of organ functions and the relation between different organs [9,10,11]. Bovard et al. established a liver/lung model, which can be used to assess the toxicity of drugs or aerosols to the human lung system [12]. Theobald et al. built a liver/kidney model to evaluate the metabolic response and toxicity of two drugs (Aflatoxin B${}_{1}$ and Benzoalphapyrene) [13]. Esch et al. used a body model composed of gastrointestine, liver, fat, kidney and bone marrow for the evaluation of the interaction between ingested nanoparticles and human tissues [14]. Oleaga et al. developed a four-organ model of the heart, liver, skeletal muscle, and nervous system, which can function well for 28 days and allow for the monitoring of mechanical and electrical activities of different cells in the long term [15].

Compared with traditional 2D cell culture, the culture of cells and tissues in a 3D environment is crucial for decent organ-on-a-chip models, since 3D culture can mimic the in vivo environment more closely by providing cell-to-cell and cell-to-extracellular matrix (ECM) communication [16]. With the advancement of microfabrication technologies, it is possible to build delicate 3D structures to mimic the in vivo environment. Gumuscu et al. made a microfluidic 3D culture platform with around 500 hydrogel compartments, which was used to investigate interaction between human intestine cells and intestinal bacteria in a high-throughput way [17]. Marsano et al. cultured cardiac cells in a 3D matrix by fibrin gel and successfully generated micro-engineered cardiac tissues with physiological functions [18]. Shim et al. made 3D villi scaffolds for the culture of Caco-2 cells to mimic the human intestine, and found that cells in 3D culture showed a more active metabolism [19]. Lee et al. used cell-printing technology to develop a 3D ECM microenvironment for a liver model [20]. Huang et al. used gelatin methacryloyl (GelMA) to build a 3D porous hydrogel structure and established an alveoli model on a chip based on the 3D hydrogel [21]. Dornhof et al. integrated 3D cell cultures and multiple electrochemical chemo- and biosensors on a microfluidic chip, and monitored culture conditions and multiple metabolites [22].

Microwells are commonly used as separated chambers for the formation and culture of 3D cell aggregates (spheroids, organoids, and embryoids) [23,24,25]. These 3D cell aggregates (spheroids, organoids, and embryoids) can help researchers to better understand cancer growth, organogenesis, and disease progression in the human body [26]. Lee et al. used poly(ethylene glycol) (PEG) hydrogel microwells to form tumor spheroids for drug screening [27]. Fukuda et al. formed hepatocyte spheroids in a microwell array, which can be used to evaluate drug metabolism efficiently [28]. Hu et al. used hydrophobic microwells to generate lung cancer organoids, which were used to predict the patient’s response to specific anti-cancer drugs [29]. Lee et al. cultured kidney organoids in microwells and provided shear stress using microfluidic flow, which was proved to be able to increase organoids’ vascular structures [30]. Karp et al. generated embryoid bodies in PEG microwells, and controlled their size and shape by adjusting the geometry of microwells [31]. Moeller et al. improved embryoids’ homogeneity by optimizing the microwell materials, cell seeding procedures and retrieval methods [25]. In general, the spheroids, organoids, and embryoids formed in these microwells have a diameter between 100 and 1000 µm. However, the microwells fabricated by traditional methods have a cuboid or cylinder shape, which is not optimized for the formation and culture of cell aggregates. Therefore, researchers have been seeking new approaches for concave microwell fabrication.

Reviews of microwell fabrication and its applications in different aspects of biomedical engineering have been provided by other researchers [32,33,34]. In this paper, we present a review of the fabrication methods of concave microwells specifically, and discuss their advantages or limitations. We will also review the applications of microwells in modeling different organs, and briefly discuss their further applications in micro-tissue engineering.

## 2. Fabrication of Concave Microwells

### 2.1. Photoresist Reflow

**Figure 1:**
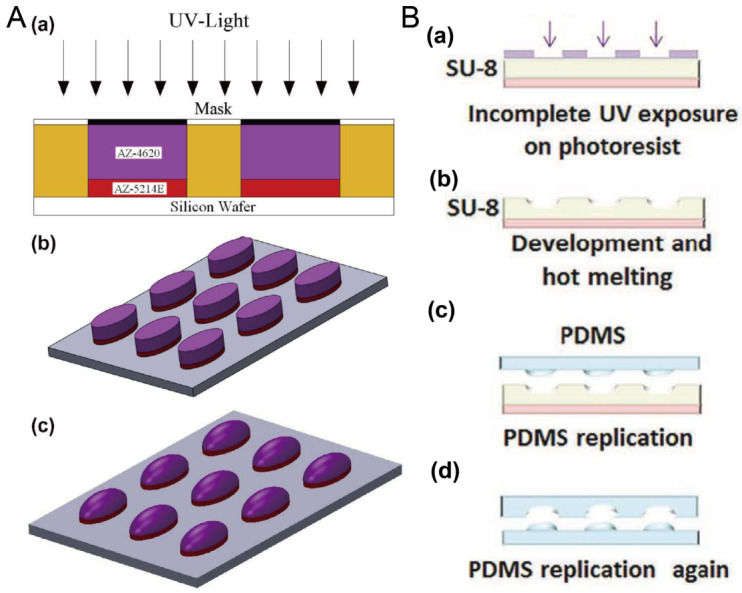
Thermal reflow of photoresist for fabrication of concave microwells. (**A**) Thermal reflow of photoresist AZ4620: at first AZ4620 and AZ5214E were patterned on a silicon wafer using standard photolithography (a,b); then, AZ4620 was melted by heating to form a convex profile (c) [35], which can be used to fabricate concave microwells with PDMS replica molding. Reprinted (adapted) from [35]. Copyright (2014) with permission from Elsevier. (**B**) The thermal reflow of photoresist SU-8 3035: at first, SU-8 3035 was patterned on a glass substrate (a), and then melted by heating (b). After that, it can be used as a mold to fabricate the concave microwells using PDMS double-replication (c,d) [37]. Reprinted (adapted) from [37]. Copyright (2015) with permission from John Wiley and Sons.

Researchers have often used photoresists AZ and SU-8 to fabricate the mold for microfluidic chips using photolithography. Here, the thermal reflow of AZ and SU-8 is utilized to prepare molds for concave microwells. After the polymer photoresist was patterned on the silicon or glass substrate using standard photolithography, it can be melted at a high temperature to form a convex or concave profile. Then, it can be used as a mold to fabricate concave microwells or microchannels using polydimethylsiloxane (PDMS) replica molding. Photoresists AZ and SU-8 are compatible with this protocol [35,36,37], as shown in Figure 1. This protocol is traditional and mature, but cleanroom facilities are necessary.

### 2.2. Lithography and Etching

**Figure 2:**
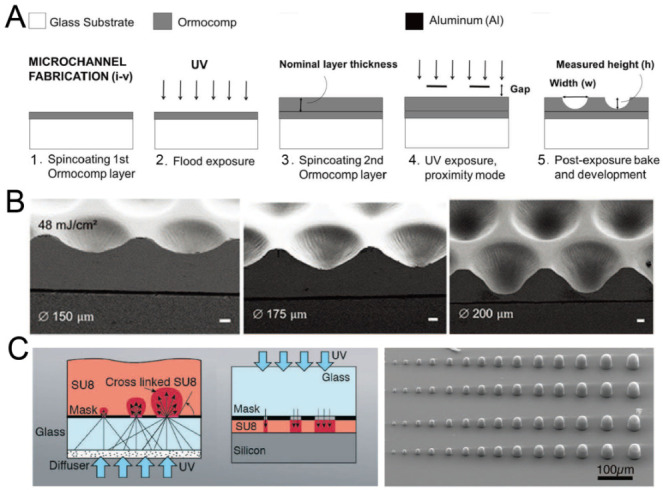
Lithography in proximity mode or backside lithography for the fabrication of concave microwells. (**A**) UV exposure of polymer Ormocomp^®^ in proximity mode: first, a thin layer of Ormocomp^®^ was coated on a glass substrate; then, another Ormocompe^®^ layer was coated and exposed to UV in proximity mode. After development, concave microwells were formed [38]. Reprinted (adapted) from [38]. Copyright (2017) with permission from AIP Publishing. (**B**) SEM pictures of concave microwells fabricated by the method described in (**A**) [39]. Scale bars: 20 µm. Reprinted (adapted) from [39] under the terms of the Creative Commons CC BY license. (**C**) The schematic view of SU-8 backside lithography: a glass diffuser was used, together with a glass mask, to diffuse the collimated UV light and crosslink dome-like SU-8 microstructures [40]. Reprinted (adapted) from [40]. Copyright (2019) with permission from the Royal Society of Chemistry.

By adjusting the exposure setup or mode, it is possible to fabricate concave microwells with single-step photolithography. Bonabi et al. fabricated concave microwells using the inorganic–organic hybrid polymer Ormocomp^®^ with UV exposure in proximity mode [38], as shown in Figure 2A. The dimensions of these concave microwells can easily be adjusted by controlling the exposure dose and gap between the mask and Ormocomp^®^ [38,39], as shown in Figure 2B. With an optical diffuser, SU-8 domes can be fabricated using backside lithography [40], as shown in Figure 2C. It is also possible to control the dimensions of SU-8 domes by adjusting the exposure dose. After that, PDMS replicas with concave microwells can be fabricated using the SU-8 molds.

**Figure 3:**
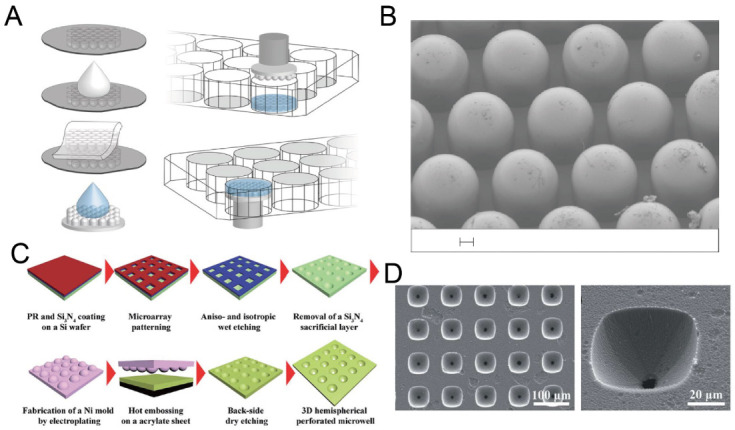
Dry etching and wet etching for the fabrication of concave microwells. (**A**) Fabrication of concave microwells on hydrogel using a silicon mold [41]. First, a silicon mold with concave microwells was used for PDMS casting. Then, the PDMS piece was used as a stamp for molding hydrogel to make concave microwells on hydrogel. (**B**) An SEM picture of the PDMS stamp. Scale bar: 100 µm. Reprinted (adapted) from [41]. Copyright (2020) with permission from Springer Nature. (**C**) The procedures of an array of perforated concave microwells using dry etching and wet etching [42]. (**D**) The SEM pictures of concave microwells using the method illustrated in (**C**) [42]. Reprinted (adapted) from [42]. Copyright (2014) with permission from Royal Society of Chemistry.

As a widely used technique for microfabrication, etching has often been used for the fabrication of concave microwells. By combining Bosch etching and lithography, silicon U-shaped microwells can be fabricated, and used to make concave microwells on hydrogel, as shown in Figure 3A,B [41]. Dry etching methods are well-developed, but require cleanroom facilities. Wet etching can be also used for the fabrication of concave microwells (such as the example shown in Figure 3C,D) [42,43,44], and generally has lower requirements for the equipment or experimental conditions.

### 2.3. Surface Tension Methods

**Figure 4:**
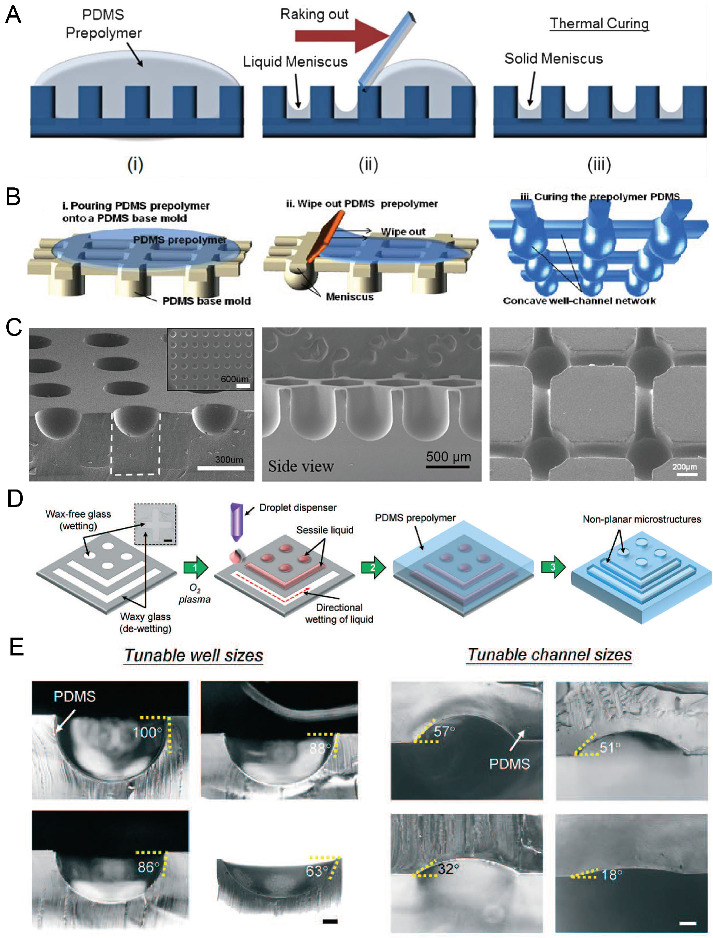
Examples of concave microwell fabrication using surface tension methods. (**A**) PDMS prepolymer was poured on a cured PDMS plate with cylinder microwells, and raked out to form a solid meniscus [46]. Reprinted (adapted) from [46]. Copyright (2013) with permission from Royal Society of Chemistry. (**B**) PDMS prepolymer was poured on a cured PDMS plate to form concave microfluidic well-channel networks [47], and (**C**) SEM pictures of concave microwells and fluidic networks using this method [45,47,50]. Reprinted (adapted) from [47]. Copyright (2014) with permission from Springer Nature. Reprinted (adapted) from [45]. Copyright (2012) with permission from John Wiley and Sons. Reprinted (adapted) from [50] under the terms of the Creative Commons Attribution License. (**D**) Liquid was patterned on glass with wax patterns, and covered by a PDMS prepolymer to form concave wells and channels [53]. (**E**) Concave wells and channels with different dimensions are fabricated using the method described in (**D**) [53]. Scale bar (left): 200 µm, scale bar (right): 100 µm. Reprinted (adapted) from [53]. Copyright (2018) with permission from Royal Society of Chemistry.

According to Young’s Laplace equation, when placing a liquid drop on a flat substrate, the profile of the liquid drop will be convex above the substrate. When placing a liquid drop into a hydrophilic microwell, the liquid profile will be concave. By using these properties, we can fabricate concave microwells in a facile way. As in Figure 4A,B, a PDMS plate with cylinder microwells is fabricated using soft lithography, PDMS prepolymer is filled into these microwells and the excess PDMS is raked out. Due to the surface tension, the remaining PDMS will have a meniscus profile. After being fully cured, the concave microwells will be formed on the PDMS plate [45,46,47,48,49,50,51,52], as shown in Figure 4C. Kuo et al. patterned liquid on the hydrophilic regions of a glass substrate, and used it as a mold for PDMS casting [53], as shown in Figure 4D. This method can be used to fabricate concave microwells and microchannels with different dimensions by controlling the contact angle of the liquid sample, as shown in Figure 4E. This method can also be used to fabricate such microstructures using other polymers, such as Off-Stoichiometry Thiol-Ene (OSTE) [54]. Bao et al. used poly-acrylic acid (PAA) solution as a sacrificial ink, and inkjet-imprinted it on precured PDMS [55]. The interaction between these two immiscible liquids can be used to fabricate concave microwells and microchannels [55]. Usually, the concave microwells fabricated using this method have a smooth surface since the contact face between two liquid phases is smooth. However, the repeatability of this method is lower than other methods since some key steps are handled with manual operation.

### 2.4. Replica Molding of Frozen Droplets

**Figure 5:**
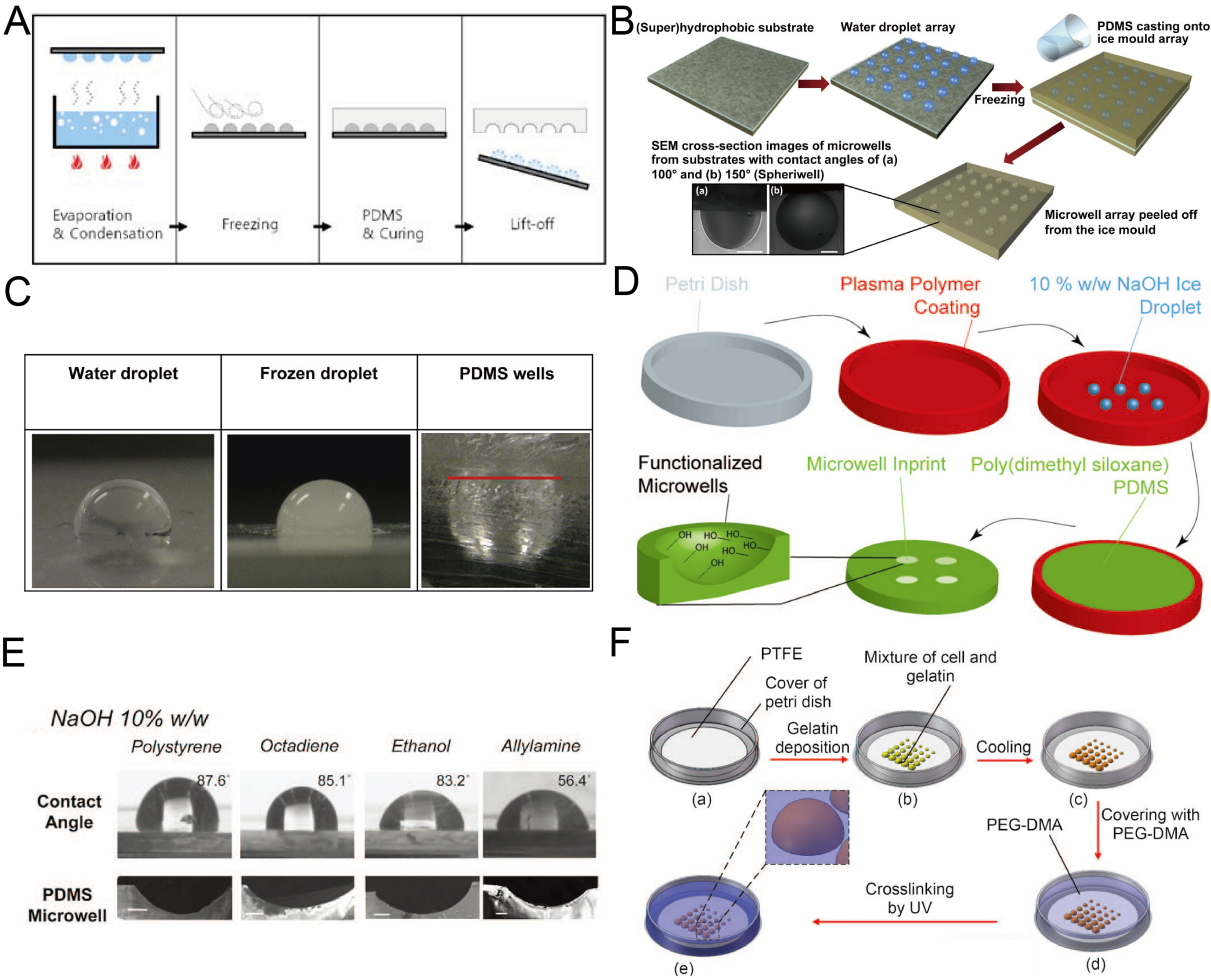
Replica molding of frozen liquid droplets. (**A**) Water vapor was condensed on a rigid substrate to form droplets. PDMS was poured on the substrate after the droplets were frozen. PDMS with concave microwells was ready after curing and lift-off [56]. Reprinted (adapted) from [56]. Copyright (2008) with permission from Springer Nature. (**B**) A water droplet array was printed on a superhydrophobic substrate and frozen, and then used as a mold for PDMS replica molding [57]. (**C**) Pictures of a water droplet, frozen droplet and the corresponding wells using the method illustrated in (**B**) [57]. Reprinted (adapted) from [57]. Copyright (2014) with permission from Elsevier. (D) 10% *w*/*w* NaOH solution was deposited on a petri dish substrate with plasma polymer coating, frozen and used as a mold for PDMS replica molding to fabricate concave microwells [58]. (**E**) Concave microwells with different dimensions were formed on different substrates using the method described in (D) [58]. Scale bars: 200 µm. Reprinted (adapted) from [58] under the terms of the Creative Commons CC BY license. (**F**) A mixture of cell and gelatin was printed on a petri dish substrate, and cooled down to form hydrogel. Then, the substrate with a hydrogel array was used to mold UV-curable PEG-DMA to obtain concave microwells [59]. Reprinted (adapted) from [59] under the terms of the Creative Commons CC BY license.

Although it is easy and fast to fabricate concave microwells using surface tension methods, sometimes it is difficult to fix the liquid pattern on the substrate, since it may be pushed away due to gravity or surface tension. Researchers developed methods for concave microwell fabrication by the replica molding of frozen liquid droplets. The frozen droplets will act as a rigid mold, similar to an SU-8 mold. The shape of the droplets can be adjusted by controlling the surface hydrophobicity. Therefore, concave microwells can be fabricated with different dimensions. Park et al. condensed water vapor on a rigid substrate, froze the water droplets and used them for PDMS replica molding [56], as shown in Figure 5A. The method can be used to achieve massive microwell fabrication. Using the same working principle, Liu et al. patterned a droplet array on a hydrophobic substrate using a non-contact spotting system and used it for microwell fabrication [57], as shown in Figure 5B,C. The contact angle of the droplet (which can be as large as 150${}^{\circ }$) can be controlled by surface hydrophobicity and the volume of the droplet can be controlled by the spotting system [57]. Functionalized PDMS microwells can be prepared when frozen NaOH solution (10%) is used as the template for replica molding [58], as shown in Figure 5D,E. Ling et al. used a mixture of gelatin and cells to build a hydrogel array to mold a UV-curable polymer, PEG-dimethacrylate (PEG-DMA) [59], as shown in Figure 5F. In addition to PDMS and PEG-DMA in the previous examples, we believe that this technique is also compatible with some other thermocurable and photocurable polymers.

### 2.5. Replica Molding of Air Bubbles

**Figure 6:**
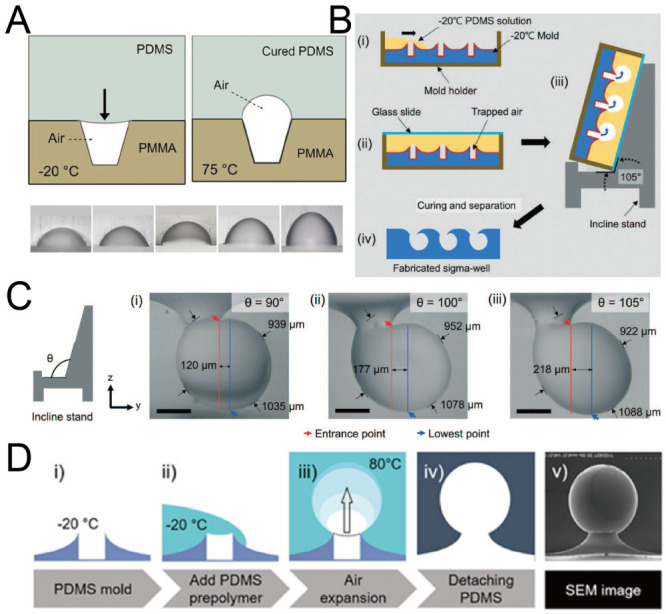
Replica molding of air bubbles. (**A**) Micro-cavities were fabricated on a PMMA base, and PDMS was poured on it. With heating, the volume of air bubbles increased and PDMS cured at the same time [60]. Reprinted (adapted) from [60]. Copyright (2012) with permission from AIP Publishing. (**B**) Low-temperature PDMS was poured onto a volcanic mountain-like PDMS mold, and placed on an inclined stand. Air bubbles expanded and PDMS cured at the same time to form Sigma-shaped microwells [62]. (**C**) Pictures of Sigma-shaped microwells using inclined stands of different angles [62]. Scale bar: 350 µm. Reprinted (adapted) from [62]. Copyright (2021) with permission from Royal Society of Chemistry. (**D**) The procedures of fabrication of Omega-shaped microwells (i–iv), and an SEM image of a microwell (v) [61]. Reprinted (adapted) from [61]. Copyright (2018) with permission from IOP Publishing.

When an air bubble is trapped in liquid, the shape of the bubble is similar to a sphere due to surface tension. Researchers have used this phenomenon to fabricate concave microwells by PDMS [60,61,62]. At first, a polymethyl methacrylate (PMMA) or PDMS base with micro-cavities was fabricated. Then, PDMS was poured onto the base, with air trapped in these micro-cavities. The air trapped in micro-cavities expanded under a high temperature and PDMS was cured at the same time. Concave microwells with different diameters and aspect ratios can be fabricated using this protocol [60], as shown in Figure 6A. It is possible to fabricate Omega-shaped or Sigma-shaped microwells by adjusting the topography of the base surface or the position of the base during curing [61,62], as shown in Figure 6B–D. Although this protocol can be used to fabricate microwells with complex shapes, it has some tricky steps, which require careful handling of the experimental setup.

### 2.6. Replica Molding of Microbeads

**Figure 7:**
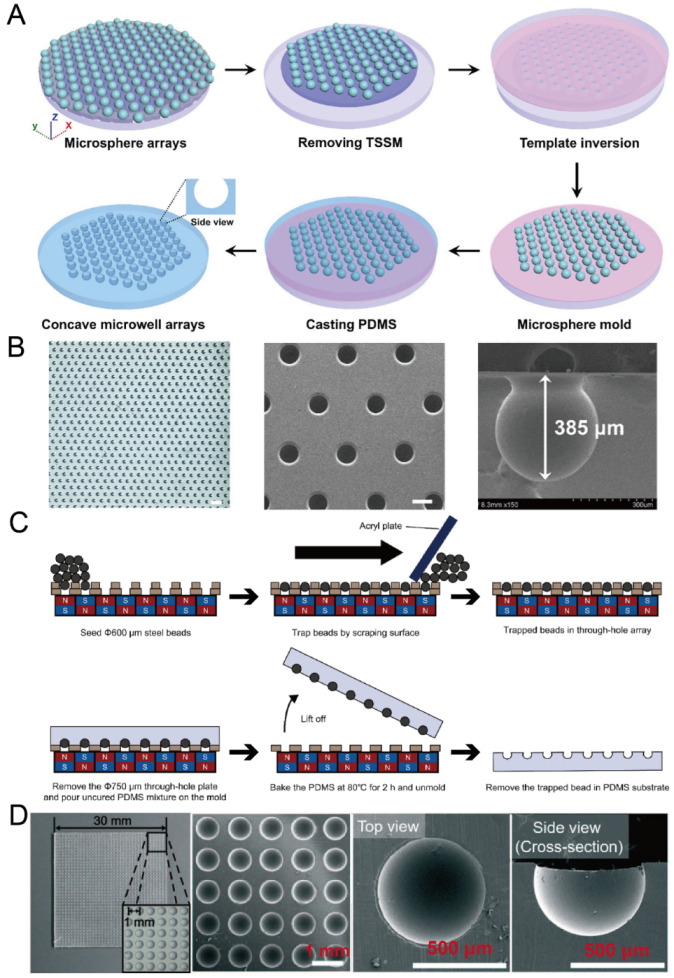
Replica molding of micro-beads. (**A**) At first, a microsphere array was formed on a glass plate utilizing a through-hole stainless steel mesh and dual adhesive tape; then, it was used for the replica molding of PDMS [63]. (**B**) A picture of a PDMS piece with concave microwells (left), an SEM picture of concave microwells (middle), and an SEM picture of a single microwell (right) [63]. The scale bar in the left picture is 1500 µm, and scale bar in the middle picture is 300 µm. Reprinted (adapted) from [63]. Copyright (2020) with permission from John Wiley and Sons. (**C**) At first, a magnetic bead array was formed using a through-hole plate under a magnetic field. After that, PDMS was poured, cured and lifted from the bead array. After removing the magnetic beads, concave microwells were ready on PDMS [65]. (**D**) Pictures of concave microwells in different magnifications prepared by the method illustrated in (**C**) [64]. Reprinted (adapted) from [64]. Copyright (2016) with permission from Royal Society of Chemistry.

Microbeads are very suitable for the molding of concave microwells because of their 3D sphere shape. To ensure the uniform distribution of microwells, researchers need to form a regular pattern of microbeads on a flat substrate. Li et al. used through-hole stainless steel meshes with dual adhesive tapes to prepare a regular microbead array [63], as shown in Figure 7A,B. Lee et al. used a through-hole plate together with a magnetic field to form a regular microbead array [64,65], as shown in Figure 7C,D. After a regular microbead array was ready on a solid substrate, it could be used for PDMS soft lithography to obtain concave microwells.

### 2.7. Deformation of Soft Membranes

**Figure 8:**
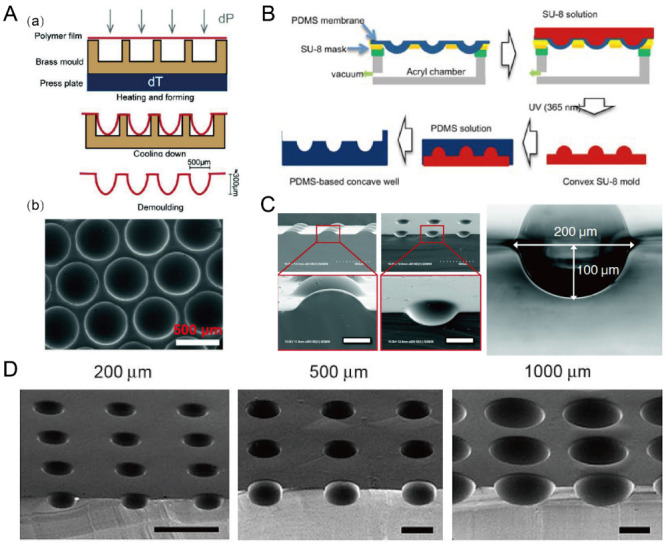
Concave microwells fabricated by methods utilizing deformed PDMS membranes. (**A**) A thin polycarbonate film was deformed by pressure and heat, and then was cooled down and demoulded (a) to obtain an array of concave microwells (b) [68]. Reprinted (adapted) from [68] under the terms of the Creative Commons Attribution-NonCommercial-NoDerivs License. (**B**) The fabrication of convex SU-8 mold: at first the PDMS membrane was deformed by vacuum, and then SU-8 was filled on the membrane and crosslinked. After that, the convex SU-8 mold was used to prepare PDMS replicas with concave microwells [71]. Reprinted (adapted) from [71]. Copyright (2011) with permission from Springer Nature. (**C**) SEM pictures of the convex SU-8 mold and concave microwells fabricated by a similar method as that described in (**B**) [69]. Scale bars: 100 µm. Reprinted (adapted) from [69]. Copyright (2009) with permission from Royal Society of Chemistry. (**D**) SEM pictures of the concave microwells of different dimensions fabricated by a similar method as that described in (**B**) [70]. Scale bars: 500 µm. Reprinted (adapted) from [70]. Copyright (2010) with permission from Elsevier.

Due to the flexibility of PDMS, thin PDMS membranes were used to assist in the fabrication of concave microwells. Nishijima et al. used a setup to prepare PDMS microwells by bending thin PDMS membranes, of which the depths can be controlled by the value of negative pressure [66]. This strategy also applies to some other polymer (such as Cyclic Olefin coPolymer (COP) and polycarbonate) films for the fabrication of concave microwells [67,68], as shown in Figure 8A. Park et al. used a similar setup to deform a thin PDMS membrane, and added SU-8 to prepare a convex SU-8 mold [69], which can be used to fabricate concave microwells of different dimensions [70,71], as shown in Figure 8B–D.

### 2.8. Laser Ablation

**Figure 9:**
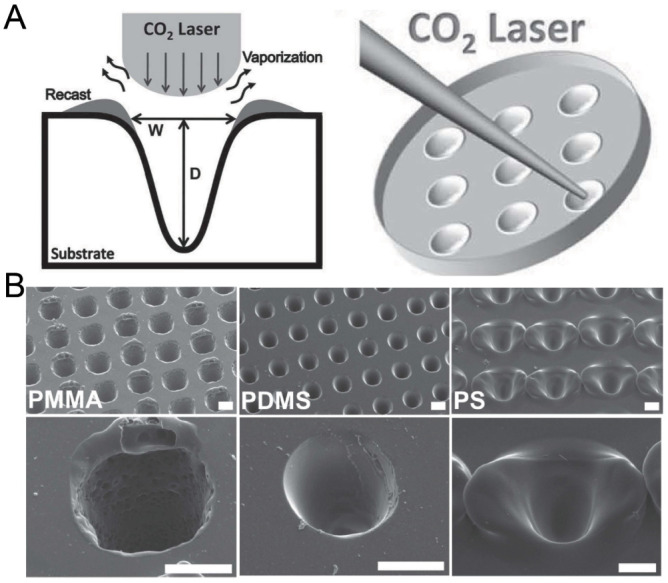
Fabrication of concave microwells by laser ablation [74]. (**A**) The schematic of the working principle of laser ablation for microwell fabrication. (**B**) The SEM pictures of concave microwells fabricated on PMMA, PDMS, and PS substrates by laser ablation. Scale bars: 100 µm. Reprinted (adapted) from [74]. Copyright (2013) with permission from John Wiley and Sons.

Laser ablation was widely used to create microstructures on polymer materials including PMMA, polystyrene (PS) and polyimide [72]. Since the energy distribution of a CO${}_{2}$ laser is similar to a Gaussian distribution [73], the laser drilling on a solid substrate can create a groove with a Gaussian profile. Tu et al. has fabricated concave microwells on a PMMA, PDMS and PS substrate using this method [74], as shown in Figure 9. The depth and shape of these microwells can be controlled by adjusting the power, pulse and focal plane position of the laser [75,76]. Although laser ablation can quickly manufacture concave microwells, the surface roughness is bigger than that of other methods (as there are usually some polymer fragments generated by the ablation remaining on the surface) and the microwell profile is more similar to a Gaussian profile than a circular arc.

### 2.9. Milling

**Figure 10:**
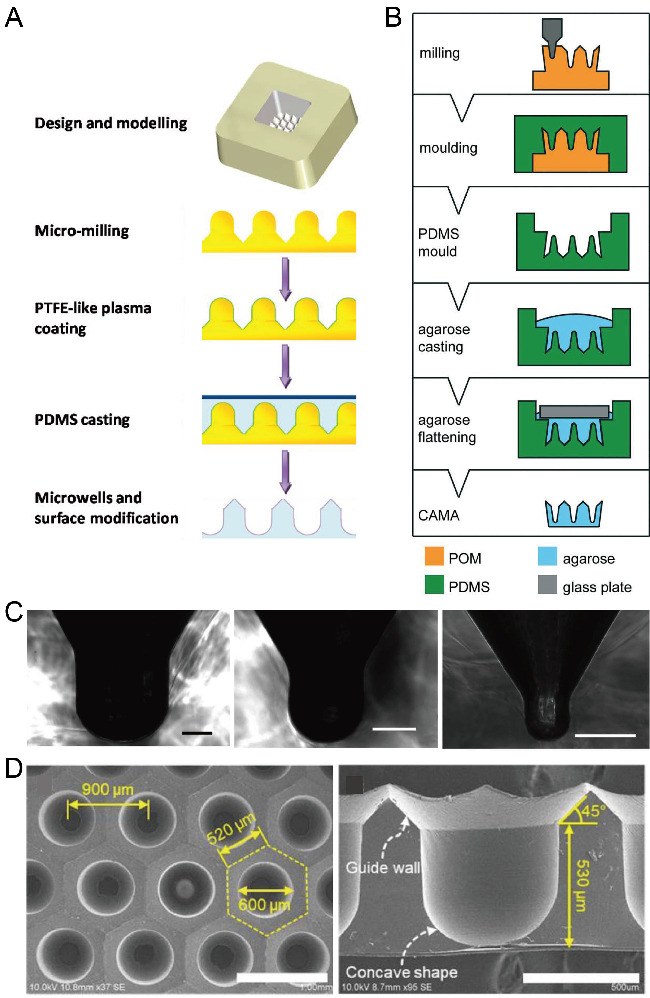
Fabrication of concave microwells by milling. (**A**) Firstly, a metallic mold was fabricated by precise CNC micromachining, and its surface was coated with polytetrafluoroethylene (PTFE)-like plasma. After that, the metallic mold was used for PDMS soft lithography to obtain the concave microwells [78]. (**B**) At first, a POM mold was prepared by CNC micromachining, and then used for double replica moldings of PDMS and agarose, respectively [81]. Reprinted (adapted) from [81]. Copyright (2018) with permission from Royal Society of Chemistry. (**C**) Pictures of a cross-section of concave microwells fabricated by the method illustrated in (A) [78]. Scale bars: 200 µm. Reprinted (adapted) from [78]. Copyright (2014) with permission from American Chemical Society. (**D**) Pictures of concave microwells fabricated by a similar method as that described in (**B**) [82]. The scale bar in the left picture shows 1 mm, and scale bar in the right picture shows 500 µm. Reprinted (adapted) from [82] under the terms of the Creative Commons CC BY license.

CNC milling is an option for the large-scale fabrication of polymer microfluidic chips at a low cost [77]. With a high-precision drilling head, CNC milling can be used to fabricate a mold with microstructures with a convex dome or concave bottom. Using these structures as a mold for single casting or double casting, researchers successfully fabricated concave microwells on PDMS or agarose substrate. Metal (such as aluminum alloy) or polymer (such as PMMA) molds with micro-dome-like structures can be fabricated by advanced CNC milling, and then used for the single casting of PDMS to obtain concave microwells [78,79,80], as shown in Figure 10A,C. Polymer (such as polyoxymethylene (POM), acrylic, or PMMA) molds with microstructures having a concave bottom were fabricated by precise CNC milling, and then used for double casting to obtain concave microwells on PDMS or agarose base [81,82,83], as shown in Figure 10B,D. CNC milling can be used to fabricate such molds with much higher efficiency compared to photolithography. However, limited by the resolution of milling itself, the surface of the mold obtained by milling is not as smooth as that obtained by other techniques (such as photolithography), which will influence the surface roughness of microwells.

### 2.10. 3D Printing

**Figure 11:**
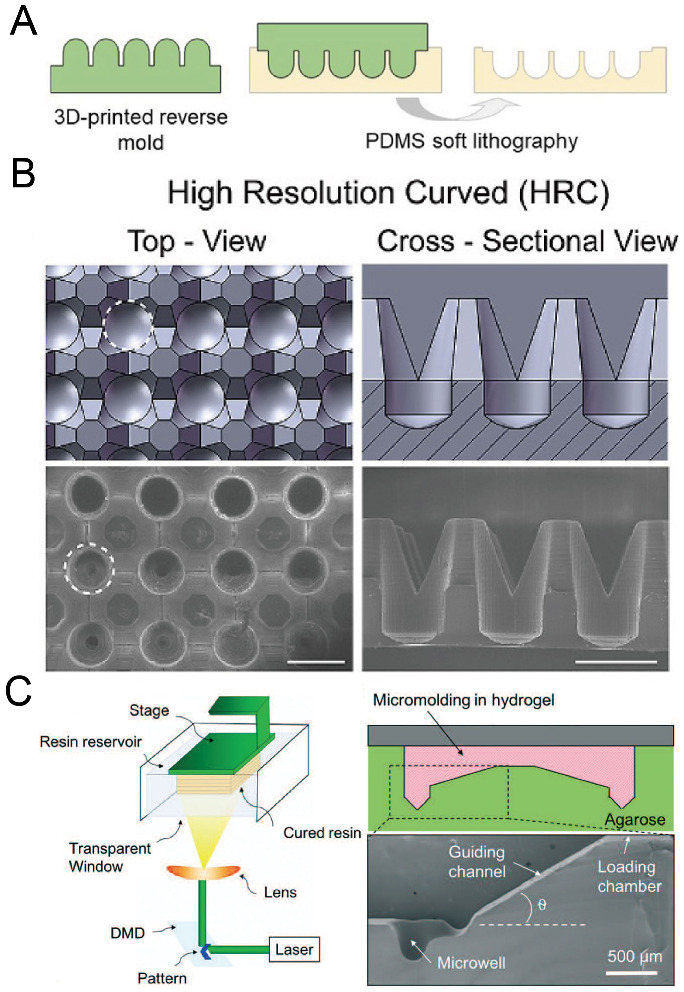
3D printing for the fabrication of concave microwells. (**A**) At first a rigid mold was prepared by 3D printing and then used for PDMS soft lithography to fabricate concave microwells [86]. (**B**) CAD views (upper) and SEM images (lower) of the concave microwells fabricated by the method illustrated in (**A**) [86]. Scale bars: 1 mm. Reprinted (adapted) from [86] under the terms of the Creative Commons CC BY-NC-ND license. (**C**) At first a mold was fabricated by 3D printing of a photocurable resin, and then the mold was used for the replica molding of agarose gel [87]. Reprinted (adapted) from [87]. Copyright (2018) with permission from Royal Society of Chemistry.

With the development of 3D printing technology, the resolution of 3D printing has become comparable with traditional lithography technology. Moreover, there are a variety of materials for choosing, and some of them are suitable for biomedical applications. 3D printing has been extensively used for constructing scaffolds with complex shapes and 3D patterning cells or tissues [84,85]. Chen et al. used direct light processing 3D printing and inkjet 3D printing to fabricate a solid mold for the following replica molding of PDMS [86], as shown in Figure 11A,B. Seyfoori et al. used dynamic light projection 3D printing to fabricate a mold with a photocurable resin, which was used to mold microwells in agarose gel [87], as shown in Figure 11C. The molds can be fabricated quickly, and it is also possible to achieve a fast iteration of mold design using 3D printing. A decent investment in the 3D printer is needed if researchers want to obtain delicate microwells with low surface roughness.

Table 1 provides a summary of the methods used for concave microwell fabrication. The majority of these methods need to use a bulky machine, such as a lithography machine, etching system, CNC milling machine, 3D printer or droplet dispenser. For the materials of mold, various kinds of materials are used depending on the specific methods, including polymer (such as photoresist, resin and PMMA), metal and glass. For the materials of microwells, PDMS is the most popular, followed by agarose.

## 3. Applications of Concave Microwells in Micro-Tissue Engineering

### 3.1. Formation of Spheroids, Organoids and Embryoids

**Figure 12:**
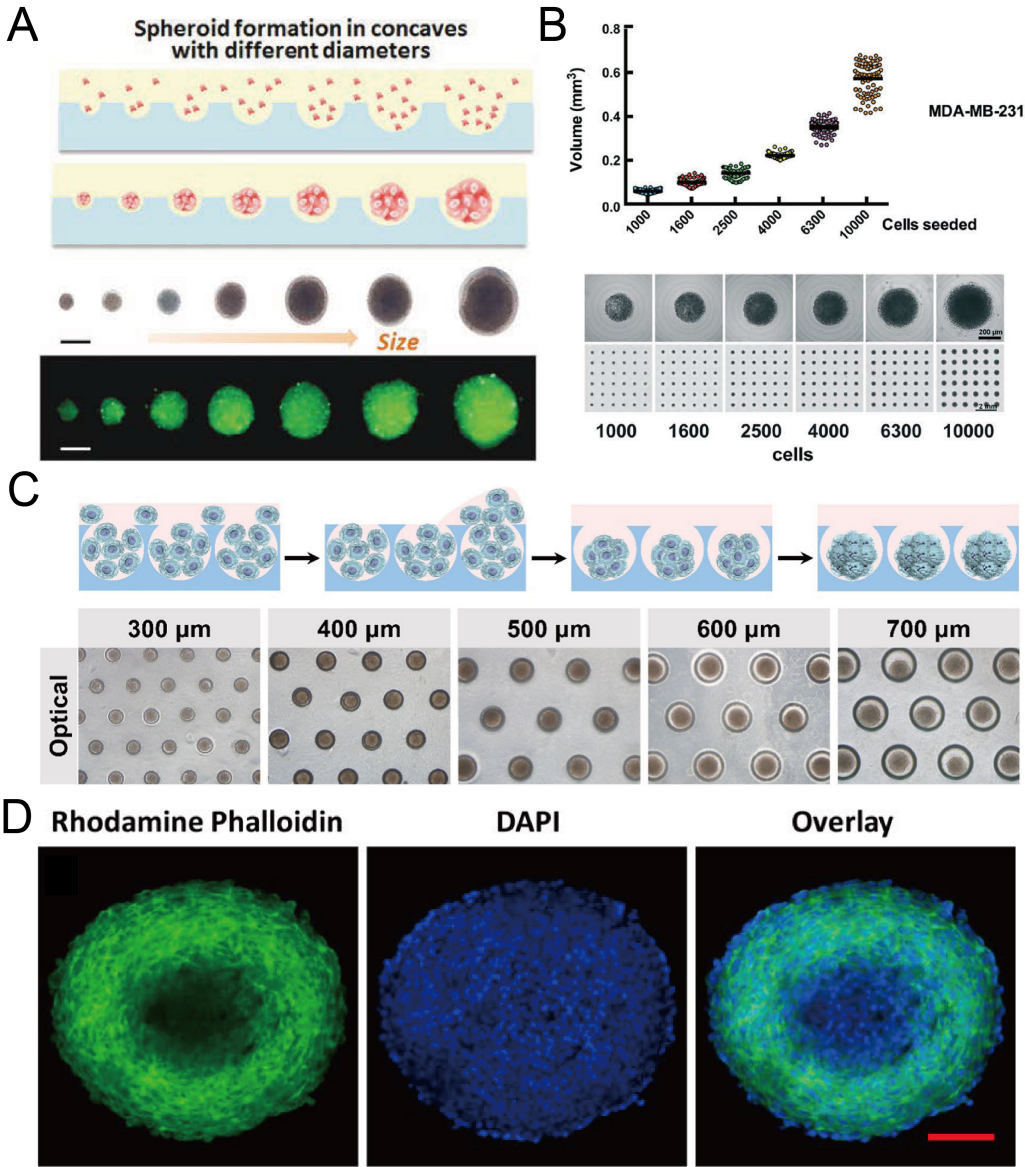
Formation of cancer spheroids in concave microwells. (**A**) Glioma spheroids of different diameters can be formed in concave microwells of different sizes [37]. Reprinted (adapted) from [37]. Copyright (2015) with permission from John Wiley and Sons. (**B**) The size of breast cancer spheroids can be controlled by the seeding density (of MDA-MB-231 cells) [81]. Reprinted (adapted) from [81] under the terms of the Creative Commons CC BY license. (**C**) Schematic view of HepG2 spheroid formation, and experimental pictures of HepG2 spheroids formed in concave microwells of different diameters [63]. Reprinted (adapted) from [63]. Copyright (2020) with permission from John Wiley and Sons. (**D**) Fluorescent images of an EMT-6 (mammary carcinoma cell line) spheroid of diameter above 400 µm, stained by DAPI and rhodamine phalloidin [78]. Scale bar: 100 µm. Reprinted (adapted) from [78]. Copyright (2014) with permission from American Chemical Society.

**Figure 13:**
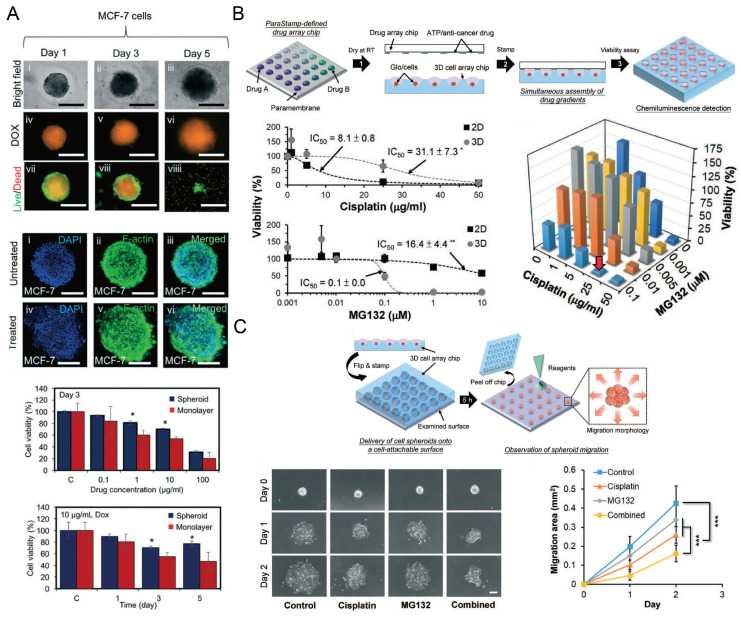
Cancer spheroids for drug screening. (**A**) The status of MCF-7 (human breast adenocarcinoma cells) spheroids was monitored after they were treated with doxorubicin (DOX). The size of these spheroids was monitored by a bright field microscope, and the uptake of DOX and the live/dead status of MCF-7 cells were monitored by a fluorescent microscope. After 3 days of DOX treatment, spheroids with nucleus and F-actin stained were compared with control groups. The influences of DOX concentration and treating time on the cell viability of spheroids and monolayers were investigated [87]. * *p* < 0.05. Scale bars: 500 µm. Reprinted (adapted) from [87]. Copyright (2018) with permission from Royal Society of Chemistry. (**B**) The schematic view of applying a 2D drug combination to SK-N-DZ (human neuroblastoma cell line) spheroids (upper), the influence of cisplatin and MG132 (a kind of proteasome inhibitor) on the cell viability of 2D and 3D culture mode (left below) respectively, and the influence of drug combination on the cell viability in spheroids after 24-h treatment (right below) [53]. ** *p* < 0.01. (**C**) The schematic view of investigating SK-N-DZ spheroid migration after drug treatment (upper), and the experimental pictures of SK-N-DZ spheroids with different drug treatments (control, cisplatin alone, MG132 alone, and a combination of cisplatin and MG132) (left below), and a comparison of the migration rate (right below) [53]. *** *p* < 0.001. Scale bar: 200 µm. Reprinted (adapted) from [53]. Copyright (2018) with permission from Royal Society of Chemistry.

**Figure 14:**
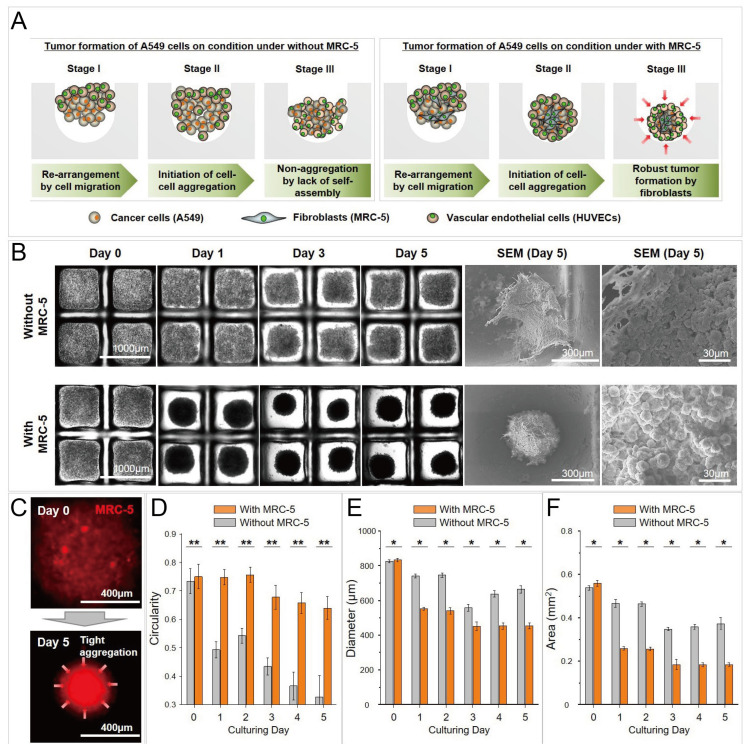
Cancer spheroids formed by co-culture of A549 (human lung cancer cells) and MRC-5 (fibroblasts), and tri-culture of A549, MRC-5, and human umbilical vein endothelial cells (HUVECS) [89]. (**A**) The schematic view of the formation of cancer spheroids by co-culture and tri-coculture. (**B**) Bright-field pictures and SEM pictures of cancer spheroids by co-culture and tri-culture in concave microwells. (**C**) Fluorescent pictures of cell aggregates, in which the MRC-5 cells were labeled by a red cell tracker. Comparison of circularity (**D**), diameter (**E**) and area (**F**) of cancer spheroids by co-culture and tri-culture. * *p* < 0.05 and ** *p* < 0.005. Reprinted (adapted) from [89] under the terms of the Creative Commons Attribution License.

Concave microwells were often used to form cancer spheroids. When cancer cells are seeded into concave microwells, of which the surfaces resist cell adhesion, cancer cells will adhere to each other and form a multicellular aggregate (cancer spheroid). Cancer spheroids can be used as a more suitable model than the 2D cell monolayer for investigating the cell behavior and drug screening. The size of cancer spheroids can be controlled by the seeding amount of cells and the size of concave microwells [37,63,81], as shown in Figure 12A–C. Traditional tools (bright field microscope and fluorescent microscope) can be used to monitor the status of spheroids, and cancer spheroids can be stained with fluorophores to observe the cell cytoskeleton [37,63,78,81], as shown in Figure 12D. After cancer spheroids are formed, they can be used to screen a single drug or a combination of different drugs [53,87,88,89,90]. The effect of the drugs can be reflected by the viability, cytoskeleton, and migration rate of cells in the spheroids [53,87], as shown in Figure 13. During cancer metastasis, the interaction between cancer cells and other types of cells in the micro-environment is important. Lee et al. generated cancer spheroids by co-culture of human lung cancer cells and vascular endothelial cells, and tri-culture of human lung cancer cells, vascular endothelial cells and fibroblasts [89], as shown in Figure 14. It was found that, with fibroblasts, cell aggregates tended to form rounder and tighter spheroids [89]. Chen et al. formed cancer spheroids using a co-culture of mouse hepatoma cells (Hepa1-6) and mouse hepatic stellate cells (JS-1), and found that the expression of transforming growth factor beta (TGF-β1) of spheroids by co-culture was higher than that by mono-culture of Hepa1-6 [90]. Moreover, the expression of alpha-smooth muscle actin (α-SMA) of spheroids by co-culture was significantly higher than that by mono-culture of JS-1 [90].

**Figure 15:**
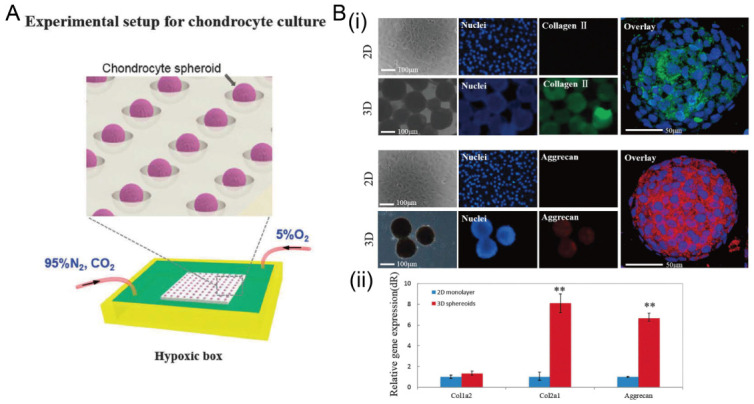
The formation of chondrocyte spheroids under a hypoxia environment in concave microwells. (**A**) Schematic view of the experimental setup for chondrocyte spheroid culture. (**B**) Fluorescent images of chondrocyte cells in 2D and 3D culture mode (i), and the comparison of gene (collagen II, collagen I, and aggrecan) expression in different culture modes (ii) [91]. ** *p* < 0.01. Reprinted (adapted) from [91]. Copyright (2015) with permission from Oxford University Press.

**Figure 16:**
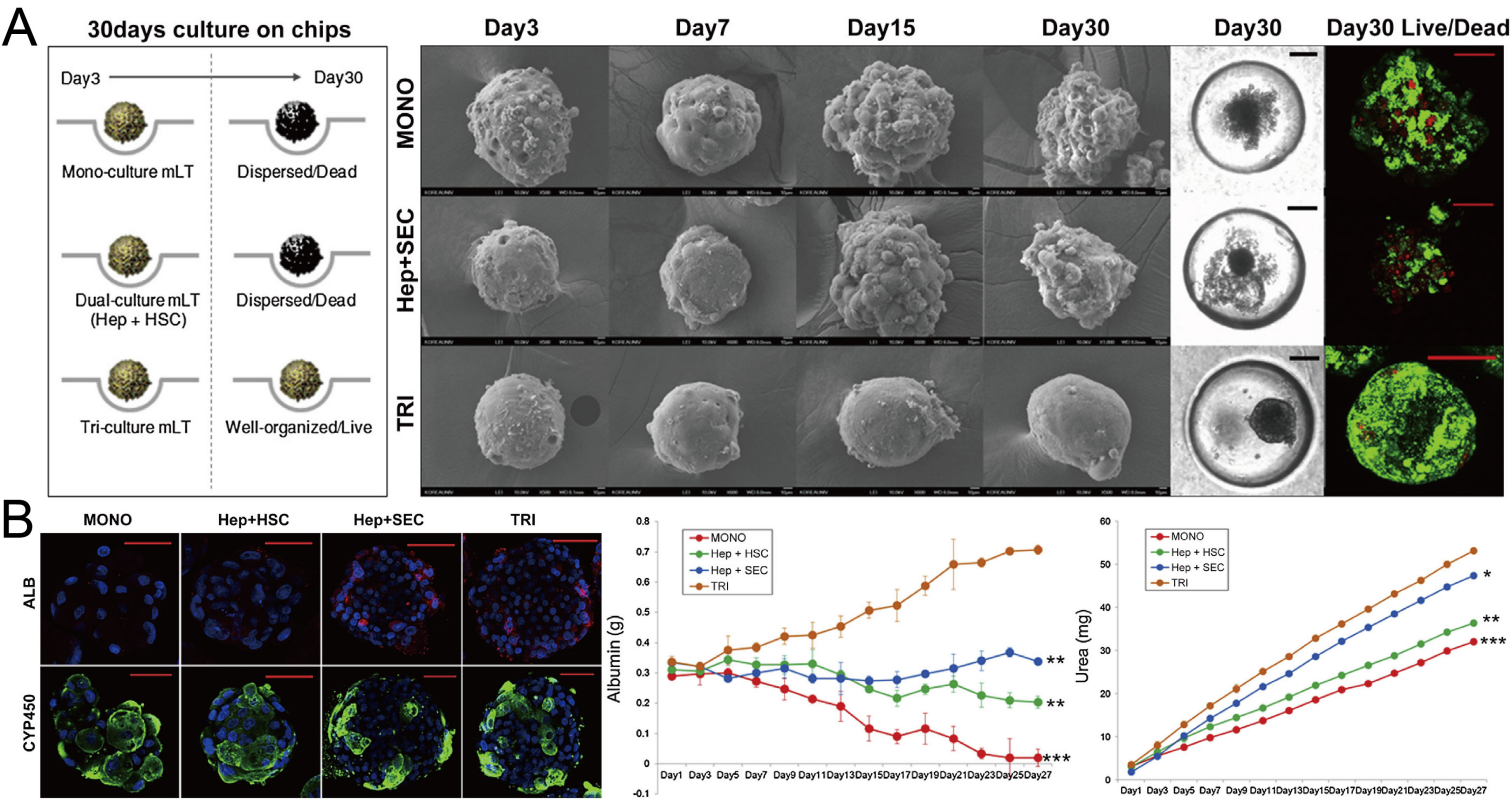
Formation of hepatic spheroids by different culture modes, and the comparison of their secretion of albumin and urea [92]. (**A**) Culture of spheroids by mono-culture of hepatocytes, co-culture of hepatocytes and hepatic stellate cells, tri-culture of hepatocytes, hepatic stellate cells and sinusoidal endothelial cells over time. Black scale bars: 100 µm, red scale bars: 50 µm. (**B**) Different spheroids with serum albumin (red), CYP450 reductase (green) and nuclei (blue) stained (left), and the secretion of albumin (middle) and urea with time (right). * *p* < 0.05, ** *p* < 0.01, *** *p* < 0.001. Scale bars: 50 µm. Reprinted (adapted) from [92]. Copyright (2014) with permission from Elsevier.

**Figure 17:**
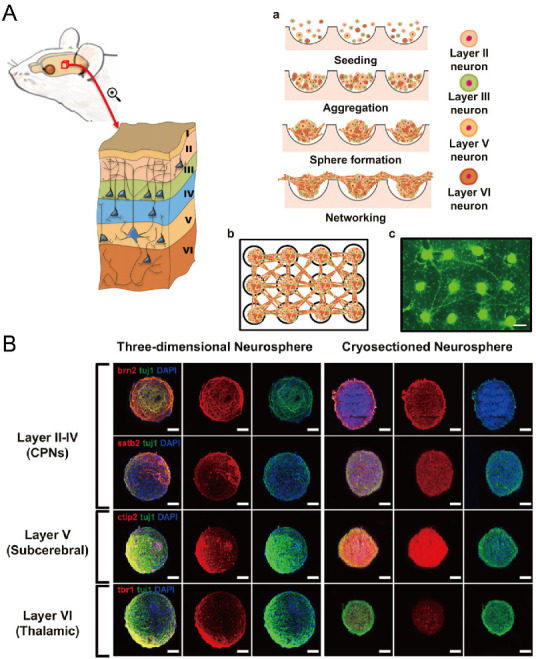
Formation of neurosphere and neural network [94]. (**A**) The schematic view showing the different layers of cortical region of prenatal rat (left), the procedures of neurosphere formation in concave microwells (right: a), the schematic of the network of neurospheres (right: b), and the pictures of neurospheres stained with calcein AM (right: c). Scale bar: 300 µm. (**B**) Fluorescent pictures of three-dimensional neurosphere and cryosectioned neurosphere stained against various transcription factors. Different transcription factors are specific to different layers of the cortical region: Brn2 and Satb2 corresponded to layers II-IV, CTIP2 corresponded to layer V, and Tbr1 corresponded to layer VI. Scale bar: 100 µm. Reprinted (adapted) from [94]. Copyright (2013) with permission from Elsevier.

**Figure 18:**
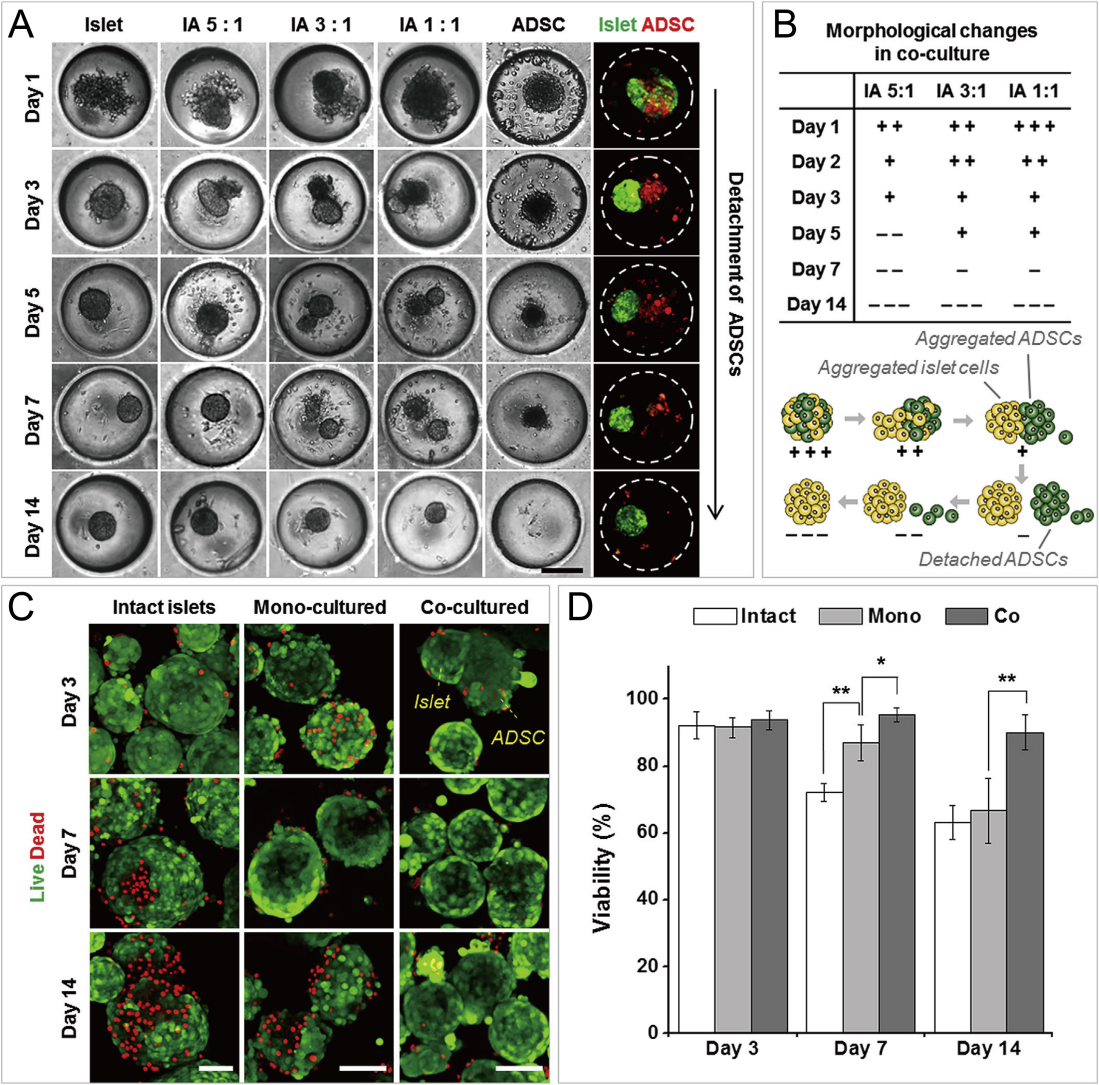
Formation of functional islet spheroids by co-culturing islet cells and ADSCs [96]. (**A**) Bright-field and fluorescent pictures of cell aggregates by mono-culture and co-culture of islet cells and ADSCs. Scale bar: 200 µm. (**B**) Morphology change of co-cultures of islet cells and ADSCs (upper) which is indicated by ’+’ and ’−’, and the schematic of the detachment of ADSCs from cell aggregates of islet cells and ADSCs (lower). (**C**) Intact islets and spheroids formed by mono-culture and co-culture, which were stained by live/dead assay. Scale bars: 100 µm. (**D**) Comparison of cell viability of intact islets and spheroids by mono-culture and co-culture. * *p* < 0.01, ** *p* < 0.001. Reprinted (adapted) from [96]. Copyright (2014) with permission from Elsevier.

**Figure 19:**
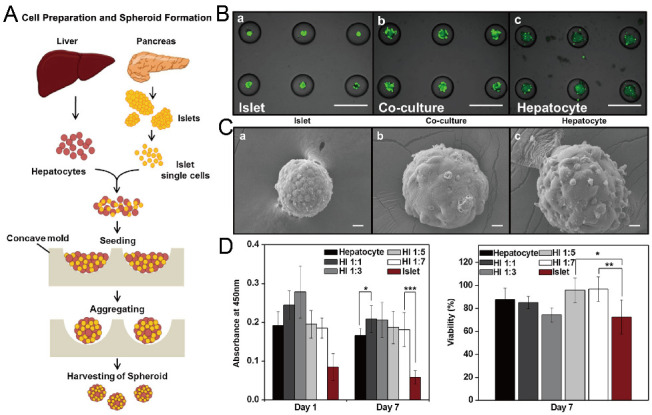
Formation of hybrid spheroids by culturing hepatocytes and islet single cells together [97]. (**A**) The schematic view of the formation of hybrid spheroids by hepatocytes and islet single cells. (**B**) Pictures of mono-culture of islet single cells (left), hepatocytes (right) and co-culture of islet single cells and hepatocytes (middle), in which the green fluorescence indicates the live cells. Scale bars: 500 µm. (**C**) SEM pictures of spheroids of mono-culture of islet single cells (left), hepatocytes (right) and co-culture of islet single cells and hepatocytes (middle). Scale bars: 20 µm. (**D**) Results of Cell Counting Kit-8 (CCK-8) applied to spheroids with different mixing ratios of hepatocytes and islet single cells on day 1 and day 7 (left), and normalized viability of cells in different spheroids on day 7 versus day 1 (right). * *p* < 0.05, ** *p* < 0.01, *** *p* < 0.001. Reprinted (adapted) from [97]. Copyright (2013) with permission from Elsevier.

In addition to cancer spheroids, researchers have also used concave microwells for the generation of spheroids by cells from various organs. Shi et al. formed chondrocyte spheroids in PDMS microwells under a hypoxia environment, and compared the gene expression of collagen I, collagen II and aggrecan of cells in 2D and 3D culture mode [91], as shown in Figure 15. The expressions of collagen I and aggrecan of 3D spheroids were significantly higher than those of 2D monolayer [91]. No et al. formed four hepatic spheroids using the mono-culture of hepatocytes alone, dual-culture of hepatocytes and hepatic stellate cells, dual-culture of hepatocytes and sinusoidal endothelial cells, and tri-culture of hepatocytes, hepatic stellate cells and sinusoidal endothelial cells [92]. Albumin and urea secretion were used as indicators of hepatic functions [92,93]. It was found that spheroids formed by tri-culture secreted the most albumin and urea while spheroids formed by mono-culture secreted the least [92], as shown in Figure 16. Chen et al. formed human cerebral organoids using human embryonic stem cells (hESCs; H9), which showed advanced characteristics, including wrinkling, lumens and neuronal layers [86]. Choi et al. generated neurospheres by co-culturing neuronal cells from four different layers of the cortical region of prenatal rats, and these neurospheres can mimic the in vivo cerebral cortex with different horizontal layers [94], as shown in Figure 17. Jeong et al. formed neural spheroids with a neural bundle connecting them, and characterized the function of the bundle by applying electrical stimulation [49]. Neurospheres formed in concave microwells have the potential to serve as an in vitro model of brain diseases, such as Alzheimer’s disease [94,95]. Jun et al. generated purified islet spheroids by co-culturing islet single cells and adipose-derived stem cells (ADSCs) [96]. With the time of co-culturing, ADSCs gradually detached from the spheroids and the final islet spheroids formed had higher viability and more secretion of insulin than spheroids by mono-culture [96], as shown in Figure 18. Lee et al. investigated the influence of oxygen permeability on the formation of pancreatic islet spheroids, and showed that spheroids formed with sufficient oxygen supply had a better performance on the stability, viability and hormone secretion [51]. Spheroids by cells from different organs with a close relationship can be also generated in concave microwells. Jun et al. formed spheroids by culturing hepatocytes and islet single cells together, and found that cells in spheroids formed by co-culture had a higher viability than cells in spheroids formed by mono-culture [97], as shown in Figure 19.

**Figure 20:**
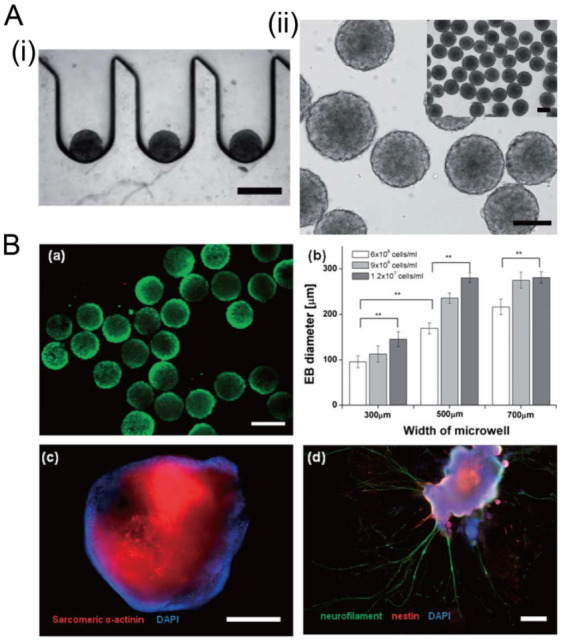
Formation of embryoid bodies in deep concave microwells [98]. (**A**) Cross-section view of embryoid stem cell aggregates in concave microwells (i), and top view of embryoid stem cell aggregates (ii). Scale bars: 400 µm. (**B**) Fluorescent images of embryoid bodies with green indicating the live cells (a), the relation of embryoid body size with the width of microwell and the seeding density (b), fluorescent images of embryoid bodies with sarcomeric a-actinin and nucleus stained to show the cardiac differentiation (c), fluorescent images of embryoid bodies with neurofilament, nestin, and nucleus stained to show the neuroepithelial differentiation (d). ** *p* < 0.01. Scale bar in (a) shows 300 µm, and scale bars in (c) and (d) show 400 µm. Reprinted (adapted) from [98]. Copyright (2012) with permission from Royal Society of Chemistry.

**Figure 21:**
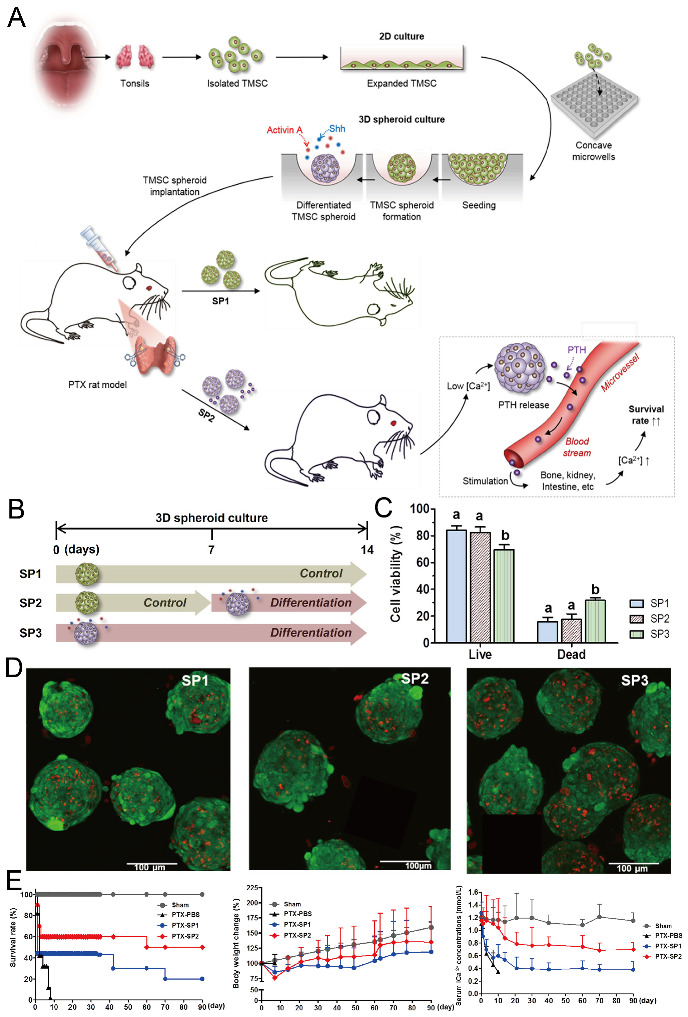
Formation of tonsil-derived mesenchymal stem cells (dTMSC) spheroids and their application in parathyroid tissue engineering [100]. (**A**) The schematic view of experimental procedures: at first, TMSCs were isolated from tonsils and then cultured in concave microwells; secondly, three different types of TMSC spheroids were formed under different culture conditions; thirdly, two types of TMSC spheroids were implanted into PTX rats to check their potential in hypoparathyroidism. (**B**) Three different types of TMSC spheroids: SP1 refers to the spheroids formed by culturing TMSCs in control medium for 14 days, SP2 refers to the spheroids formed by culturing TMSCs in control medium for the first 7 days and differentiation medium for the second 7 days, and SP3 refers to the spheroids formed by culturing TMSCs in differentiation medium for 14 days. (**C**) The viability of TMSCs in SP1, SP2, and SP3, respectively. (**D**) Fluorescent pictures of SP1 (left), SP2 (middle), and SP3 (right) stained by a live/dead assay, in which the green indicates the live cells and the red indicates the dead cells. (**E**) The comparison of survival rate (left), body weight change (middle) and serum iCa${}^{2+}$ concentration (right) of PTX rats implanted with SP1 and SP2 in 90 days. PBS served as a negative control. Sham refers to rats with sham operation but no spheroid implantation.

**Figure 22:**
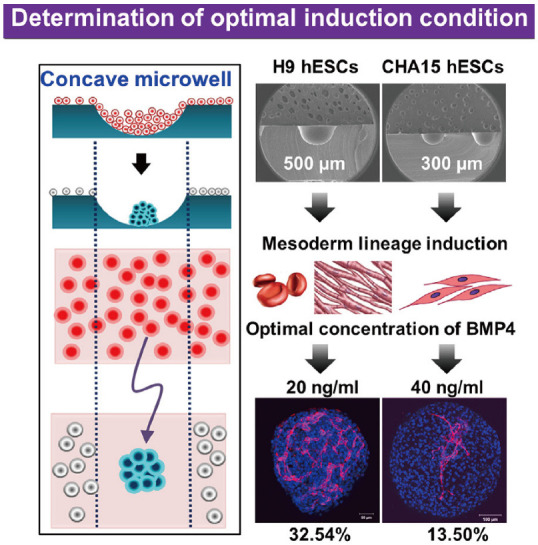
Formation of embryoid bodies and procedures of determination of optimal induction condition [48]. At first, hESCs were seeded into concave microwells to form embryoids. Then, these embryoids were treated by mesoderm inducer BMP4 after mesoderm lineage induction. Lastly, the distribution of platelet endothelial cell adhesion molecule (PECAM) was checked to evaluate the effect of BMP4.

Concave microwells can also be used to generate stem cell spheroids by 3D culturing stem cells. Researchers have generated embryoid bodies using embryoid stem cells in concave microwells of different dimensions, before harvesting them and studying their differentiation by staining specific proteins [45,98,99], as shown in Figure 20. Lee et al. formed spheroids by culturing human adipose-derived stem cells (hASCs) and studied their differentiation by checking the gene expression related to chondrogenic differentiation [64]. The relevant gene expression of cells in spheroids is significantly higher than that of cells by 2D culture [64]. Park et al. generated different types of spheroids by culturing tonsil-derived mesenchymal stem cells (TMSCs), and implanted them in parathyroidectomized (PTX) rats to study their applications in parathyroid tissue engineering [100], as shown in Figure 21. It was found that PTX rats with differentiated spheroids implanted had a higher survival rate than that of PTX rats with undifferentiated spheroids. The optimization of differentiation conditions (size of microwells and concentration of mesoderm inducer BMP4) of H9- and CHA15-human embryonic stem cells (hESCs) was achieved [48], as shown in Figure 22.

From the previous examples about spheroids/organoids/embryoids, generally cells by 3D culture can show more physiologically relevant characteristics than cells by 2D culture, which is closer to those of the in vivo organs. Therefore spheroids/organoids/embryoids formed in concave microwells have a high potential for use in drug screening and theranostics. As shown in Table 2, cancer spheroids are mostly generated for drug screening or studies of irradiation effects. Organoids are usually harvested for xenogeneic implantation or used as a model for some specific disease. Researchers often use embryoids to investigate differentiation conditions.

### 3.2. Study of Cellular Behavior

**Figure 23:**
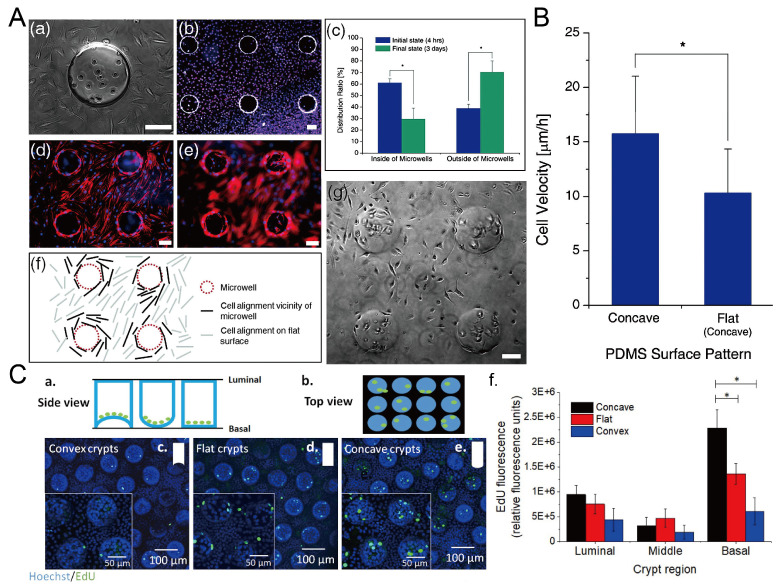
The behavior of cells in concave microwells. (**A**) 4 h after the seeding of L929 cells on the PDMS substrate with concave microwells (a), fluorescent images of L929 cells after three days of culture (b), the distribution of cells inside and outside of concave microwells in 4 h and 3 days after seeding (c), fluorescent images of human mesenchymal stem cells (hMSCs) cultured on PDMS substrate with concave microwells, of which the focus plane is on the flat plane (d) and concave microwells (e) respectively, the pattern of cells close to the microwells (f), and the status of L929 cells after three-day culture on the substrate with concave microwells (g) [69]. * *p* < 0.01. Scale bars: 100 µm. (**B**) Comparison of cell velocity on concave and flat surfaces [69]. ‘*’ indicates a significant difference (*p* = 0.034). Reprinted (adapted) from [69]. Copyright (2009) with permission from Royal Society of Chemistry. (**C**) Schematic side view (a) and top view (b) of primary human colonic epithelial cell culture in microwells with different shapes, fluorescent images of cells cultured on different bases (c–e), and the comparison of 5-ethynyl-2${}^{\prime }$-deoxyuridine (EdU) fluorescence of cells cultured on different bases (f) [102]. * *p* < 0.001. Reprinted (adapted) from [102]. Copyright (2021) with permission from IOP Publishing.

The surface topography of the culture substrate has an influence on the behavior of cells growing on it, including the viability, alignment, and migration velocity [101]. Park et al. found that L929 mouse fibroblast cells preferred to grow on the flat surface, and the cells inside the microwells showed a decline after three-day culture [69], as shown in Figure 23A,B. The migration velocity of cells in concave microwells is higher than cells cultured on flat surfaces [69]. Howard et al. cultured primary human colonic epithelial cells on collagen microwells with flat, concave, and convex bottom surfaces, and found that curvature of the culture substrate had an effect on the cell proliferation [102], as shown in Figure 23C.

## 4. Conclusions

We briefly summarized the current methods used to fabricate concave microwells, as well as their applications in micro-tissue engineering. Among these options for microwell fabrication, some need to have cleanroom facilities or expensive tools/equipment, while some do not. Researchers can choose a suitable method according to their available lab resources for fabrication. In addition, the materials of microwells also need to be considered from the perspectives of fabrication and application. In general, there is an obvious trend that the location of concave microwell fabrication is transferring from professional microfabrication labs to common biochemical labs. Similarly, the equipment or machines used for fabrication are changing from bulky microfabrication tools in cleanroom to benchtop tools. Such a transition will facilitate the application of concave microwells in micro-tissue engineering and provide convenience to the end-users. For applications in micro-tissue engineering, concave microwells have already found successful applications in spheroid/organoid/embryoid formation and cellular behavior study. For future applications, on the one hand, more multiorgan-on-a-chip models will be built using the spheroids/organoids/embryoids formed in concave microwells, and on the other hand, more analyses at the molecular level will be integrated into the 3D culture platform. Furthermore, the spheroids/organoids/embryoids harvested from these culture platforms will find more applications in drug screening and xenogeneic implantation. We believe that spheroid/organoid/embryoid formation in concave microwells and their following applications in theranostics will last as a hot research topic for a while.

## Figures and Tables

**Table 1:** Summary of fabrication methods for concave microwells.

Fabrication Method	Equipment/Tools	Materials of the Mold	Materials of the Microwells	Ref.
Photoresist reflow	Lithography machine	AZ or SU-8 photoresist	PDMS	[35,37]
Lithography	Lithography machine	N.A.	Polymer Ormocomp	[38,39]
		SU-8	PDMS	[40]
Etching	Dry etching - Etching system	SU-8	PDMS, Hydrogel	[41]
	Wet etching - N.A.	N.A.	Glass	[43]
Surface tension methods	Lithography machine	SU-8	PDMS	[46]
	Droplet dispenser	Glass, water	PDMS	[53]
Replica molding of frozen droplets	Automated non-contact spotting system	Hydrophobic PDMS, frozen water	PDMS	[57]
	Pressure-assisted value-based bioprinting system	Petri dish, gelatin	PEG-DMA	[59]
Replica molding of air bubbles	Computer-controlled milling machine	PMMA, air bubble	PDMS	[60]
	N.A.	CPU pin array, PDMS, air bubble	PDMS	[61]
Replica molding of microbeads	Through-hole steel mesh, dual adhesive tape	Glass, microsphere array	PDMS	[63]
	Through-hole plate, magnet array	Microsphere array	PDMS	[64,65]
Deformation of soft membranes	Lithography machine	SU-8	PDMS	[69,70]
Laser ablation	CO 2 laser	N.A.	PMMA, PDMS and PS	[74]
Milling	CNC milling machine	Metal	PDMS	[78]
		POM, PDMS	Agarose	[81]
3D printing	3D printer	3D printing resin	PDMS, agarose	[86,87]

N.A. means not applicable for that method. Ref. column only provides representative citation sources.

**Table 2:** Spheroids, organoids, and embryoids by various cell lines and their applications in tissue engineering.

Spheroids, Organoids, Embryoids	Cell Lines	Applications	Ref.
Cancer spheroids	Human astrocytoma cell line U87	Drug screening (hypoxia-inducible factors(HIFs) inhibitors), the influence of hypoxia	[37]
	Pancreatic cancer cells MIA PaCa-2	Effect of combined chemotherapy (cisplatin) and irradiation	[81]
	Human liver cancer cell line HepG2	Drug screening (doxorubicin hydrochloride)	[63]
	Mammary carcinoma cell line EMT-6	Drug screening (curcumin)	[78]
	Mono-culture of human breast cancer cell line MCF-7, Mono-culture of human astrocytoma cell line U87	Drug screening (doxorubicin)	[87]
	Human neuroblastoma cell line SK-N-DZ	Combined effect of cisplatin and MG132	[53]
	Tri-culture of human lung cancer cells A549, human lung fibroblasts MRC-5, and human umbilical vein endothelial cells	Combined effect of paclitaxel + Gemcitabine, paclitaxel alone, Gemcitabine alone	[89]
	Mono-culture of mouse hepatoma Hepa1-6 cells, mono-culture of mouse hepatic stellate JS-1 cells, co-culture of Hepa1–6 cells and JS-1 cells	Drug screening (paclitaxel)	[90]
Organoids	Rat chondrocytes	Drug screening (HIFs inhibitors), the influence of hypoxia	[91]
	Mono-culture (hepatocytes alone), co-culture of hepatocytes + hepatic stellate cells (HSCs), co-culture of hepatocytes + sinusoidal endothelial cells (SECs), tri-culture (hepatocytes + HSCs + SECs)	Xenogeneic implantation	[92]
	Prenatal rat cortical neurons	Neurotoxicity study of amyloid beta	[94]
	Primary rat neural progenitor cells	Neuronal signal transmission through neurite bundles	[49]
	Mono-culture of islet single cells, co-culture of islet single cells and ADSCs	Xenogeneic implantation	[96]
	Co-culture of rat pancreatic islet cell and rat primary hepatocyte	Xenogeneic implantation	[97]
Embryoids	Murine R1 ES cell line	Validation of differentiation capabilities	[45,98]
	Human adipose-derived stem cells	Validation of differentiation capabilities	[64]
	Human tonsil-derived mesenchymal stem cells	Xenogeneic implantation	[100]
	Human embryonic stem cells (H9- and CHA15-hESCs)	Optimization of differentiation conditions	[48]

## Data Availability

Not applicable.
